# Ventilation SPECT/CT-guided air leak management: improving precision in a long-standing surgical challenge

**DOI:** 10.3389/fsurg.2026.1780031

**Published:** 2026-02-24

**Authors:** Marta Fuentes, María Teresa Gómez-Hernández, Andrea Peñaherrera Cepeda, Cristina Rivas, Jose Luis Aranda, Oscar Colmenares, Mario Manama, Felipe Gómez-Caminero, Pilar Tamayo, Marcelo F. Jiménez

**Affiliations:** 1 Service of Thoracic Surgery and Lung Transplantation, Salamanca University Hospital, Salamanca, Spain; 2 Salamanca Institute of Biomedical Research, Salamanca, Spain; 3 University of Salamanca, Salamanca, Spain; 4 Service of Nuclear Medicine, Salamanca University Hospital, Salamanca, Spain

**Keywords:** prolonged air leak (PAL), pulmonary resection, secondary spontaneous pneumothorax (SSP), surgical outcome, ventilation SPECT/CT

## Abstract

**Objectives:**

Prolonged air leak (PAL) remains a significant surgical challenge after pulmonary resections and secondary spontaneous pneumothorax. Ventilation single-photon emission computed tomography combined with computed tomography (vSPECT/CT) has emerged as a promising tool for air leak localization. This study evaluates the impact of preoperative vSPECT/CT on surgical management of PAL.

**Methods:**

This single-center study compared a prospectively enrolled vSPECT/CT cohort (February 2021–December 2025) with retrospective historical controls (January 2015–January 2021). Propensity score matching generated two matched groups: with and without vSPECT/CT. Primary outcome was vSPECT/CT accuracy in detecting air leaks; secondary outcomes included chest tube duration, persistent PAL, reoperation rates, and hospital stay. Wilcoxon signed-rank and McNemar tests were used for comparisons.

**Results:**

A total of 122 patients were included (52 vSPECT/CT; 70 non-vSPECT/CT). vSPECT/CT showed a 95.3% concordance with intraoperative air leak localization. After matching, 86 patients (43 per group) were analyzed. The vSPECT/CT group had shorter chest tube duration (median 2 vs. 4 days; *P* = 0.01) and hospital stay (median 3 vs. 5 days; *P* = 0.036). Rates of persistent PAL and reoperation were similar between groups (*P* = 0.057 and *P* = 0.375, respectively).

**Conclusion:**

In this prospectively enrolled cohort, preoperative vSPECT/CT reliably localizes PAL, enabling focused surgical intervention and supporting more efficient postoperative recovery. Its use is associated with shorter chest tube duration and hospital stay, highlighting its value as a preoperative tool in the management of PAL.

## Introduction

Prolonged air leak (PAL) remains one of the most persistent challenges in thoracic surgery, frequently occurring after pulmonary resections and in the setting of secondary spontaneous pneumothorax (SSP). Despite advances in minimally invasive techniques and perioperative care, PAL continues to be associated with increased morbidity, prolonged hospital stay, and increased healthcare costs (1–3).

According to data from the European Society of Thoracic Surgeons (ESTS) Silver Book database, the incidence of PAL after lobectomy approaches 10% (4) and 15.3% after segmentectomy, as reported in a recent study (5). Moreover, multicenter studies have identified PAL as one of the leading causes of unplanned reinterventions following anatomical lung resection, ranking second only to postoperative haemorrhage and accounting for a substantial proportion of unexpected reoperations (24.4%) (6). In line with these findings, Dezube et al. (7) reported that 16 out of 129 patients who developed PAL after pulmonary resection required surgical reintervention, indicating that approximately 12% of PAL cases fail to resolve with conservative management and ultimately necessitate operative treatment.

Several chronic pulmonary diseases are associated with an increased risk of SSP and PAL (8–11). In this context, pneumothorax represents a clinical manifestation of underlying structural lung abnormalities rather than an isolated event. In selected cases, surgical correction of the air leak is indicated; however, this approach poses a significant challenge due to the heterogeneous and fragile nature of the diseased pulmonary parenchyma (11–13).

Traditionally, surgical management of PAL relied on open thoracotomy, which, although associated with low recurrence rates, entailed significant morbidity, including increased blood loss, postoperative pain, and prolonged hospital stay (14). Consequently, video-assisted thoracic surgery (VATS) has become the preferred approach in current practice. However, despite the advantages of minimally invasive surgery, intraoperative identification of the air leak during reintervention remains technically challenging (15). Conventional techniques, such as the intraoperative water submersion test (iWST), are often difficult to apply in minimally invasive settings, where operative space is limited and adequate lung inflation may be suboptimal. These limitations can compromise precise localization of the air leak and have been associated with higher conversion rates to open surgery.

Ventilation single-photon emission computed tomography combined with computed tomography (vSPECT/CT) has recently emerged as a promising preoperative diagnostic tool for air leak localization (16–18). This technique allows the identification of focal tracer accumulation at the site of air escape, potentially enabling more accurate surgical planning.

Therefore, the present study aims to evaluate the impact of incorporating preoperative vSPECT/CT into the surgical management of patients with PAL by comparing clinical outcomes between patients managed with and without preoperative vSPECT/CT.

## Methods

### Ethical statement

The study was approved by the Clinical Research Ethics Committee (IRB code: 2021 06 802). Written informed consent was obtained from all patients prospectively included after the implementation of the preoperative vSPECT/CT protocol (February 2021). For the historical control group, the requirement for informed consent was waived by the Ethics Committee due to the retrospective nature of data collection and the exclusive use of anonymized data for analysis and publication.

### Study design, data source, and patients

This single-center comparative study with historical controls included patients with PAL secondary to pulmonary resection surgery or SSP. In the vSPECT/CT era (February 2021–December 2025), all patients referred for possible surgical management of PAL were prospectively evaluated using a preoperative vSPECT/CT protocol. In the historical control period (January 2015–January 2021), only patients who ultimately underwent surgical intervention for PAL were included. Consequently, comparative analyses were intentionally restricted to patients who received surgical treatment for PAL in both periods.

For the purposes of this study, PAL was defined as an air leak persisting for more than 5 days after lung surgery or after chest tube insertion for SSP. Eligibility for surgical evaluation required not only persistence but also a clinically significant air leak, defined as >2/5 on an analogue drainage system or >200 mL/min on a digital drainage system. Patients with lower-grade air leaks were considered suitable for conservative management and were therefore not included in the study. The historical control group comprised patients who underwent surgery for pleural air leaks prior to the introduction of vSPECT/CT, while patients managed after its implementation were prospectively enrolled. Although all patients in the vSPECT/CT era were prospectively assessed for surgical treatment, the present comparative analysis was restricted to those who ultimately underwent surgical intervention. Patients in whom surgery was deferred or avoided, either due to clinical decision-making or based on vSPECT/CT findings, were excluded from the comparative analysis, as the primary aim of the study was to evaluate the impact of preoperative vSPECT/CT on intraoperative localization and surgical outcomes.

Perioperative management was standardized throughout the study period. A structured program of early and intensive postoperative respiratory physiotherapy had been in place since 2002 (19) and was consistently applied to all patients. VATS had been fully implemented and standardized at our institution since 2014, and minimally invasive approaches were used whenever feasible. Air leak monitoring was performed using an analog drainage system throughout the study period until January 2025, when digital drainage systems were introduced. Chest drain management policy was consistent throughout the study period and consisted of removing the chest tube when no air leak was evident in the analogue drainage system (no bubbling, 0/5) or when air leak was <10 mL/min over the last 12 h recorded in the digital drainage system and fluid output was <450 mL/24 h. In accordance with institutional protocols, suction was not routinely applied to chest drains.

For the historical control group, all clinical, surgical, and pathological data had been prospectively collected at the time of care in a dedicated institutional database, ensuring data completeness and accuracy. Variables were defined according to standardized criteria from the Society of Thoracic Surgeons and the European Society of Thoracic Surgeons (20).

## vSPECT/CT protocol

Radioventilation was performed using ^99mTc-Technegas delivered via the Venticis® II system. Firing and flushing cycles were carried out according to the manufacturer's specifications using medical-grade argon. Technegas was administered with the patient in a moderately reclined position and nasal airflow occluded. Patients were instructed to perform three to five deep inhalations, each followed by a 5-second Valsalva maneuver.

vSPECT/CT acquisition was performed on a hybrid SPECT/CT system equipped with a low-energy high-resolution collimator. SPECT data were acquired using a body-contouring orbit (128 × 128 matrix, zoom 1.0, 180° rotation, 32 projections, 15 s per projection), followed by a low-dose CT scan for attenuation correction and anatomical localization. SPECT images were reconstructed using an iterative algorithm, both with and without attenuation correction, while CT images were reconstructed as 2-mm axial slices for anatomical co-registration. Image fusion and analysis were performed using dedicated workstation software.

For most patients, vSPECT/CT was performed in the days immediately preceding the planned surgery.

### Outcomes

**Figure 1:**
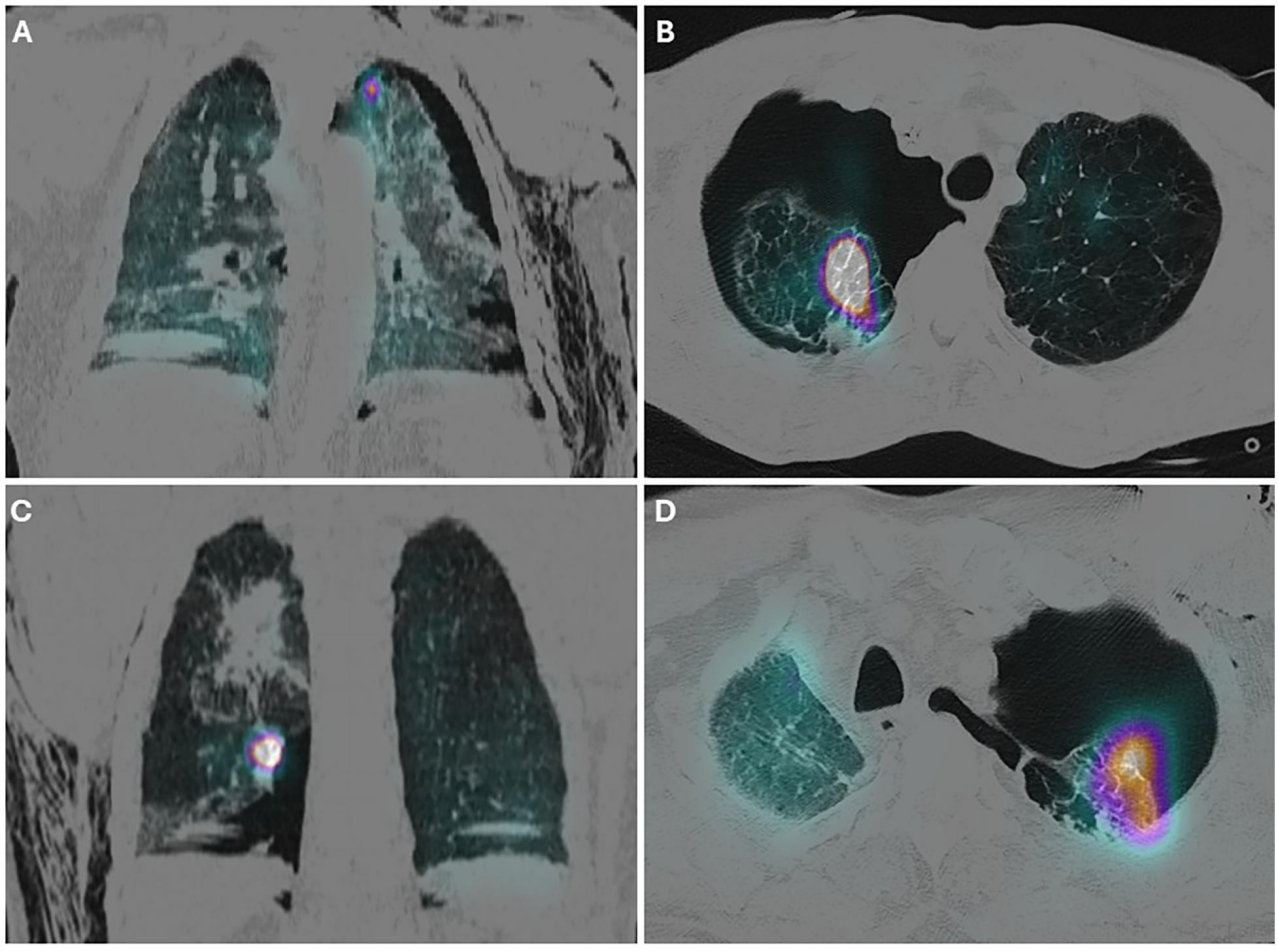
vSPECT/CT images showing coronal **(A,C)** and axial **(B,D)** slices from different patients, demonstrating leakage of 99mTc-technegas into the lung parenchyma.

Air leak sites were defined as focal areas of increased ^99mTc-Technegas accumulation within the lung parenchyma, corresponding to the presumed site of air escape (Figure 1), with localization recorded by lobe and along transverse and longitudinal axes. Scans were considered non-diagnostic if no focal tracer accumulation was observed. In cases where surgery was performed despite a negative vSPECT/CT, any operative interventions were also recorded.

Patients were divided into two groups according to the use of preoperative vSPECT/CT. The primary outcome was the concordance between vSPECT/CT–based air leak localization and intraoperative surgical findings. Concordance between vSPECT/CT localization and intraoperative findings was independently evaluated by two surgeons involved in the procedure. No blinding was applied, as the primary purpose of vSPECT/CT was to guide surgical management. Secondary outcomes included chest tube duration, used as an indirect measure of successful surgical management reflecting correct intraoperative identification and treatment of the air leak, as well as persistent PAL —defined as an air leak lasting more than 5 days following the primary surgical procedure performed to resolve PAL, regardless of its magnitude—, reoperation rates due to air leak and length of hospital stay. Reoperation due to air leak was defined as the need for a second surgical procedure specifically aimed at controlling the air leak because of failure of the initial intervention; only reinterventions related to air leak control were considered, and reoperations for other postoperative complications were excluded.

Subgroup descriptive analyses by air-leak etiology (post-resection vs. SSP) were performed for the unmatched cohort to explore potential differences in surgical outcomes.

### Statistical analysis

Baseline characteristics, clinicopathological variables and outcomes were compared between the vSPECT/CT and historical control groups. Continuous variables were expressed as mean (SD) for normally distributed data or median (IQR) for non-normally distributed data, and categorical variables as frequencies and percentages. Normality was assessed using the Kolmogorov–Smirnov test, and homogeneity of variances was evaluated with Levene's test. Normally distributed continuous variables were compared using Student's t-test, while non-normally distributed variables were compared with the Mann–Whitney U test. Categorical variables were analysed using Pearson's chi-square or Fisher's exact test, as appropriate. All tests were two-sided, and a *p*-value <0.05 was considered statistically significant.

To assess the robustness of our findings, two complementary analyses were performed:

- Primary analysis: included all eligible patients (unmatched cohort). This approach was justified by the absence of statistically significant differences in baseline characteristics between groups (all *p* > 0.1).
- Propensity score matching (PSM) analysis: performed as a sensitivity analysis. Patients were matched 1:1 using optimal matching, which minimizes the global sum of distances between matched pairs. Propensity scores were estimated with a logistic regression model including age, body mass index (BMI), radiological emphysema, and cause of prolonged air leak (lung resection vs. secondary spontaneous pneumothorax), selected *a priori* based on clinical relevance. Unmatched controls were excluded from further analyses. Covariate balance was assessed using standardized mean differences (SMD), with values <0.1 considered indicative of adequate balance (21). Postoperative outcomes were compared between matched groups using the Wilcoxon signed-rank test for continuous variables and the McNemar test for categorical variables.

All analyses were conducted using RStudio (version 4.3.2, The R Foundation, Vienna, Austria) with the “MatchIt” package version 4.5.5, and SPSS v28.0 (SPSS Inc., Chicago, IL, USA) for further data analysis. The manuscript was prepared in accordance with STROBE reporting guidelines.

## Results

A total of 122 patients were initially included: 52 in the vSPECT/CT group and 70 in the historical non–vSPECT/CT control group. The vSPECT/CT was well tolerated, and no related adverse events were observed. Nine patients (eight with SSP and one with post-resection PAL) were excluded because surgical correction of the air leak was ultimately not performed. In seven patients with SSP, the absence of a clearly detectable air leak combined with a high surgical risk led to deferral of surgery and continuation of conservative management. In the remaining patient, the air leak was localized to a parahilar region, and a non-surgical approach was therefore adopted. In all excluded cases, air leak resolution was achieved spontaneously or following chemical or autologous blood pleurodesis administered through the chest tube. In the post-resection PAL case, although an air leak was identified, it was successfully managed conservatively with autologous blood patch pleurodesis through the chest tube, obviating the need for surgical reintervention. Overall, surgical intervention was avoided in 9 of 52 patients (17.3%) initially evaluated for possible operative management in the vSPECT/CT era.

Following these exclusions, the final vSPECT/CT cohort comprised 43 patients. Baseline demographic and clinical characteristics were comparable between groups, with no clinically relevant differences observed in age, sex distribution, body mass index, or surgical indication.

**Figure 2:**
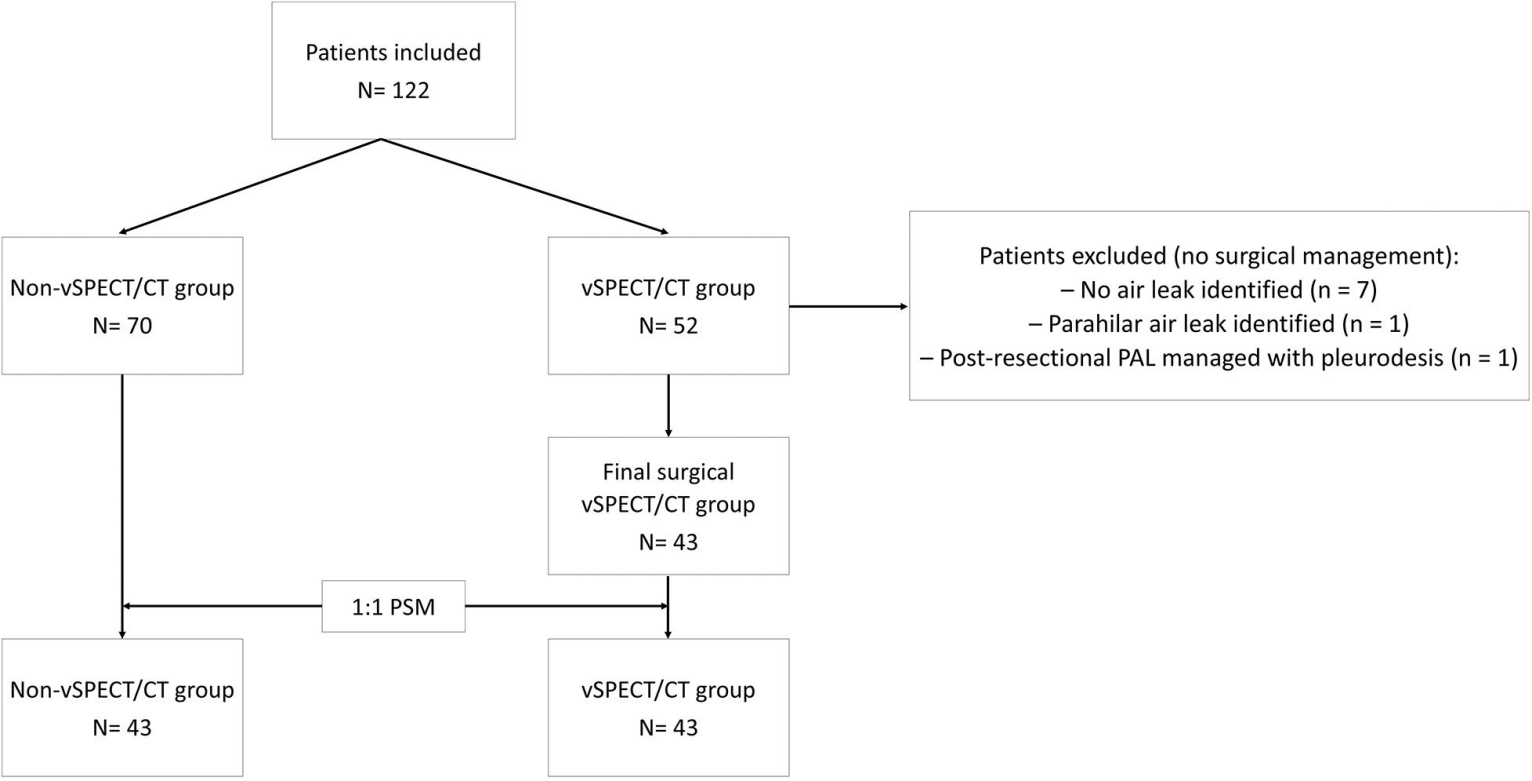
Flow diagram of patient inclusion, vSPECT/CT evaluation, surgical management, and final cohort selection.

After optimal 1:1 propensity score matching, 86 patients (43 per group) were analyzed. All covariates, including those not included in the propensity score model, achieved adequate balance, with standardized mean differences below 0.1. Only sex (SMD = 0.14) and BMI (SMD = 0.12) exceeded this threshold, but these differences were considered negligible (Table 1). Figure 2 displays the flow diagram of patient inclusion, vSPECT/CT evaluation, surgical management, and final cohort selection.

**Table 1:** Patient demographics and baseline characteristics before and after PSM.

Variable	Before PSM				After PSM			
	vSPECT/CT (*n* = 43)	Non-vSPECT/CT (*n* = 70)	SMD	*p*-value	vSPECT/CT (*n* = 43)	Non-vSPECT/CT (*n* = 43)	SMD	*p*-value
Age, median (IQR), years	64 (20)	67.5 (15)	0.120	0.642*	64 (20)	67 (18)	0.034	0.739*
BMI, median (IQR), kg/m2	24.39 (6.57)	24.01 (4.46)	0.057	0.668*	24.39 (6.57)	24.09 (3.72)	0.117	0.836*
Male gender, *n* (%)	37 (86)	64 (91.4)	0.155	0.368***	37 (86)	39 (90.7)	0.145	0.501**
Smoking history, *n* (%)	41 (95.3)	66 (94.3)	0.051	1***	41 (95.3)	41 (95.3)	<0.001	1***
Coronary disease, *n* (%)	8 (18.6)	16 (22.9)	0.109	0.592**	8 (18.6)	8 (18.6)	<0.001	1**
Diabetes, *n* (%)	4 (9.3)	5 (7.1)	0.074	0.729***	4 (9.3)	4 (9.3)	<0.001	1***
Hypertension, *n* (%)	19 (44.2)	25 (35.7)	0.171	0.370**	19 (44.2)	17 (39.5)	0.094	0.662**
Radiological emphysema, *n* (%)	27 (62.8)	36 (51.4)	0.235	0.238**	27 (62.8)	27 (62.8)	<0.001	1**
Cause of PAL, *n* (%)			0.161	0.383**			0.100	0.631**
- Lung resection	13 (30.2)	16 (22.9)			13 (30.2)	11 (25.6)		
- SSP	30 (69.8)	54 (71.1)			30 (69.8)	32 (74.4)		

PSM, propensity score matching; vSPECT/CT, ventilation single-photon emission computed tomography/computed tomography; IQR, interquartile range; PAL, prolonged air leak; SSP, secondary spontaneous pneumothorax.

* *P*-value for Mann–Whitney *U*-test.

** *P*-value for Chi-squared test.

*** *P*-value for Fisher's exact test.

### Primary outcome

Among the 43 patients in the vSPECT/CT group who underwent surgery, focal tracer accumulation was detected in 38 cases, with complete concordance between vSPECT/CT findings and intraoperative localization in all of them. The remaining five patients showed no detectable tracer accumulation on vSPECT/CT but underwent surgery because of persistent or high-volume air leak. In three of these cases, no active air leak was identified during revision surgery, and all resolved following surgical intervention using a technique that included pleurodesis. In the two remaining patients, vSPECT/CT failed to identify an air leak that was subsequently detected intraoperatively; these false-negative findings were attributed to incomplete lung expansion in the setting of PAL. Overall, vSPECT/CT demonstrated a concordance rate of 95.3% (41/43) with intraoperative findings.

Surgical approaches and procedures are summarized in Table 2. The use of a VATS approach was significantly higher in the vSPECT/CT group compared with the non-vSPECT control group (90.7% vs. 62.9%; *P* = 0.001), and this difference remained significant after matching (*P* = 0.008).

**Table 2:** Intraoperative outcomes of the study cohort before and after PSM.

Outcome	Before PSM			After PSM		
	vSPECT/CT (*n* = 43)	Non-vSPECT/CT (*n* = 70)	*p*-value	vSPECT/CT (*n* = 43)	Non-vSPECT/CT (*n* = 43)	*p*-value
VATS approach, *n* (%)	39 (90.7)	44 (62.9)	0.001*	39 (90.7)	27 (62.8)	0.008**
Surgical technique, *n* (%)			0.023*			0.007*
- Manual local repair (±pleurodesis)	8 (18.6)	15 (21.4)		8 (18.6)	9 (20.9)	
- Mechanical closure (±pleurodesis)	35 (81.4)	37 (52.9)		35 (81.4)	24 (55.8)	
- Pleural-based intervention	0 (0)	16 (22.9)		0 (0)	9 (20.9)	
- Anatomical lung resection	0 (0)	2 (2.9)		0 (0)	1 (2.3)	

PSM, propensity score matching; vSPECT/CT, ventilation single-photon emission computed tomography/computed tomography; VATS, video-assisted thoracic surgery.

* *P*-value for Chi-squared test.

** *P*-value for Mc Nemar test.

### Secondary outcomes

Patients in the vSPECT/CT group had significantly shorter chest tube duration (median, 2 vs. 3.5 days; *P* < 0.001) and shorter postoperative length of stay (median, 3 vs. 5 days; *P* = 0.002) compared with the non-vSPECT group. In addition, the prevalence of persistent PAL following surgical intervention was significantly lower in the vSPECT/CT group (11.6% vs. 28.6%; *P* = 0.035). Reoperation rates did not differ significantly between groups (2.3% vs. 7.1%; *P* = 0.405). No significant differences were observed in 30-day overall morbidity, mortality, or readmission rates.

After propensity score matching, the differences in chest tube duration (median, 2 vs. 4 days; *P* = 0.01) and postoperative length of stay (median, 3 vs. 5 days; *P* = 0.036) remained significant. No differences were observed between groups in the prevalence of persistent PAL, reoperation rates, morbidity, mortality, or readmission rates (Table 3).

**Table 3:** Outcomes in postoperative variables before and after PSM.

Outcome	Before PSM			After PSM		
	vSPECT (*n* = 43)	Non-vSPECT (*n* = 70)	*p*-value	vSPECT (*n* = 43)	Non-vSPECT (*n* = 43)	*p*-value
Chest tube duration (days), median (IQR)	2 (2)	3.5 (4.25)	<0.001*	2 (2)	4 (5)	0.01****
Overall morbidity, *n* (%)	5 (11.6)	9 (12.9)	0.847**	5 (11.6)	5 (11.6)	1*****
Persistent PAL (>5 days), *n* (%)	5 (11.6)	20 (28.6)	0.035**	5 (11.6)	13 (30.2)	0.057*****
Reoperation rate, *n* (%)	1 (2.3)	5 (7.1)	0.405***	1 (2.3)	4 (9.3)	0.375*****
Postoperative hospital stay (days), median (IQR)	3 (2)	5 (4)	0.002*	3 (2)	5 (5)	0.036****
30-day mortality, *n* (%)	1 (2.3)	1 (1.4)	1***	1 (2.3)	1 (2.3)	1*****
Readmission, *n* (%)	1 (2.3)	8 (11.4)	0.150***	1 (2.3)	3 (7)	0.5*****

PSM, propensity score matching; vSPECT/CT, ventilation single-photon emission computed tomography/computed tomography; IQR, interquartile range; PAL, prolonged air leak.

* *P*-value for Mann–Whitney *U*-test.

** *P*-value for Chi-squared test.

*** *P*-value for Fisher's exact test.

**** *P-*value for Wilcoxon signed-rank test.

***** *P*-value for Mc Nemar test.

The results of the subgroup descriptive analyses by air-leak aetiology are summarized in Table 4.

**Table 4:** Outcomes by air leak aetiology of the unmatched cohort.

Outcome	Post-resection (*n* = 29)			Spontaneous secondary pneumothorax (*n* = 84)		
	vSPECT (*n* = 13)	Non-vSPECT (*n* = 16)	*p*-value	vSPECT (*n* = 30)	Non-vSPECT (*n* = 54)	*p*-value
Chest tube duration (days), median (IQR)	2 (4.5)	5.5 (8)	0.022*	1 (1.25)	3 (3)	0.002*
Overall morbidity, *n* (%)	3 (23.1)	4 (25)	1***	2 (6.7)	5 (9.3)	1***
Persistent PAL (>5 days), *n* (%)	2 (15.4)	9 (56.3)	0.052***	3 (10)	11 (20.4)	0.222**
Reoperation rate, *n* (%)	0 (0)	2 (12.5)	0.488***	1 (3.3)	3 (5.6)	1***
Postoperative hospital stay (days), median (IQR)	4 (4.5)	6 (2.75)	0.028*	2 (1.25)	4 (4)	0.004*
30-day mortality, *n* (%)	1 (7.7)	0 (0)	0.448***	0 (0)	1 (1.9)	1***
Readmission, n (%)	1 (7.7)	4 (25)	0.343***	0 (0)	4 (7.4)	0.292***

PSM, propensity score matching; vSPECT/CT, ventilation single-photon emission computed tomography/computed tomography; IQR, interquartile range; PAL, prolonged air leak.

* *P*-value for Mann–Whitney *U*-test.

** *P*-value for Chi-squared test.

*** *P*-value for Fisher's exact test.

## Discussion

PAL remains a common and clinically significant complication following pulmonary resection and SSP, often prolonging hospital stay and increasing healthcare costs. Beyond its direct clinical implications, PAL imposes a substantial burden on patients and healthcare systems, particularly when prolonged drainage fails to achieve spontaneous resolution. Strategies that facilitate accurate localization and definitive surgical treatment of air leaks are therefore of major interest in thoracic surgery. As intraoperative localization of air leaks can be challenging, especially during VATS, preoperative functional imaging may provide added value by guiding targeted surgical intervention.

Our study demonstrates that preoperative vSPECT/CT enables reliable localization of air leaks and is associated with improved postoperative outcomes. In the prospectively enrolled vSPECT/CT cohort, imaging findings showed a high concordance with intraoperative air leak localization (95.3%), facilitating targeted surgical intervention. These findings reflect concordance between imaging-guided localization and surgical exploration rather than formal diagnostic accuracy. Unlike purely anatomical imaging, vSPECT/CT provides functional information on regional ventilation, facilitating precise identification of actively leaking lung segments. To assess the clinical impact of this strategy, we performed propensity score matching incorporating variables known to increase the risk of prolonged air leak after pulmonary resection (22). Importantly, the integration of vSPECT/CT into preoperative planning was associated with a significant reduction in chest tube duration and length of hospital stay compared with matched patients managed without functional imaging guidance.

These findings are consistent with preliminary reports by Krieger et al. (17), which suggested a potential clinical impact of vSPECT/CT, as both total surgical time and incision-to-suture time were shorter following its introduction. In our study, operative time could not be formally assessed for the historical cohort due to the unavailability of operative time data; however, the shorter postoperative course observed in the vSPECT/CT group suggests that improved preoperative localization may enhance overall procedural efficiency.

Minimally invasive approaches were also more frequently employed in the vSPECT/CT group. It should be noted, however, that historical controls were treated during an earlier phase of the institutional VATS learning curve, and the lower VATS rate in this group may reflect evolving surgical expertise rather than the absence of preoperative vSPECT/CT alone. Moreover, a higher proportion of patients in the vSPECT/CT group underwent targeted surgical interventions directed specifically at the site of the air leak. In contrast, in the non–vSPECT/CT cohort, more than 20% of patients were managed with pleural-based procedures alone, without a targeted approach to the underlying source of the air leak. In the initial unmatched cohort, persistent PAL was more frequent following intervention in the non-vSPECT/CT group, highlighting a clinically relevant difference consistent with the expected benefit of targeted leak localization. However, after propensity score matching, these differences were no longer significant, reflecting balanced comparison between groups.

These outcomes suggest that vSPECT/CT facilitates more efficient identification and management of air leaks, leading to faster postoperative recovery. This is particularly relevant in frail patients, for whom prolonged drainage and hospitalization are associated with increased morbidity.

One of the main strengths of this study is the prospective design of the vSPECT/CT cohort, which enabled a structured assessment of its impact on surgical decision-making. Importantly, in a relevant proportion of patients (7/52), vSPECT/CT showed no evidence of an active air leak, supporting a conservative management strategy. In one additional patient, vSPECT/CT localized the air leak to a parahilar region, where surgical correction was considered high risk and therefore avoided. In all these cases, the management strategy guided by vSPECT/CT was not associated with adverse outcomes or 30-day mortality. These findings suggest that vSPECT/CT provides clinically meaningful information not only by enabling targeted surgical intervention, but also by accurately identifying patients in whom surgery can be safely avoided.

Other methods for air leak localization have also been explored. In particular, aerosolized indocyanine green (ICG) inhalation has shown promising results, as it facilitates identification of air leak sites that may be missed with the conventional water submersion test (23, 24). This technique may help reduce postoperative air leaks, particularly in patients undergoing VATS, although it remains investigational and requires further validation in larger clinical studies.

The most recent European consensus (25) emphasizes the importance of a multidisciplinary approach to the management of PAL, the standardization of definitions, and the need for robust evidence-based algorithms to optimize patient outcomes. In this multidisciplinary and algorithm-based context, vSPECT/CT could potentially play a useful complementary role in the assessment and management of PAL.

Despite these encouraging results, several methodological limitations should be acknowledged. First, the absence of a diagnostic gold standard limits formal estimation of accuracy. The iWST itself is an experience-based method of unknown sensitivity and specificity, and obtaining definitive results in thoracoscopic cases remains challenging (26). Additionally, intermittent or temporarily sealed air leaks may not be evident at the time of operation, potentially leading to misclassification of some vSPECT findings. Second, the single-center design and moderate sample size may limit the generalizability of our findings. Third, although propensity score matching was applied, residual confounding due to unmeasured variables cannot be fully excluded. In particular, missing or incomplete data in the retrospective cohort—especially among patients with SPP—limited the inclusion of several clinically relevant covariates in the matching process. This limitation should be considered when interpreting the comparative outcomes.

Finally, because vSPECT/CT was used as a guidance tool during surgery, blinding between imaging findings and intraoperative assessment was not feasible, and observer bias cannot be completely excluded in the interpretation of air leak concordance. Therefore, future prospective, multicenter studies incorporating standardized intraoperative metrics and a multimodal, standardized definition of pulmonary air leak—combining complementary intraoperative techniques and structured postoperative verification—are needed to further define the role of vSPECT/CT in surgical decision-making, explore its predictive value, and refine indications and optimal timing for its use.

## Conclusion

In conclusion, preoperative vSPECT/CT is a valuable tool for localizing the origin of PAL, enabling targeted surgical intervention and more precise operative planning. Its use is associated with shorter chest tube duration and hospital stay, supporting its integration into preoperative protocols for patients in the surgical management of PAL.

## Data Availability

The raw data supporting the conclusions of this article will be made available by the authors, without undue reservation.
